# The Gut-Microbiome in Gulf War Veterans: A Preliminary Report

**DOI:** 10.3390/ijerph16193751

**Published:** 2019-10-04

**Authors:** Patricia A. Janulewicz, Ratanesh K. Seth, Jeffrey M. Carlson, Joy Ajama, Emily Quinn, Timothy Heeren, Nancy Klimas, Steven M. Lasley, Ronnie D. Horner, Kimberly Sullivan, Saurabh Chatterjee

**Affiliations:** 1 Environmental Health Department, Boston University School of Public Health, Boston, MA 02118, USA; 2 Environmental Health and Disease Laboratory, Department of Environmental Health Sciences, Arnold School of Public Health, University of South Carolina, Columbia, SC 29208, USA; 3 Biostatistics and Epidemiology Data Analytics Center, Boston University School of Public Health, Boston, MA 02118, USA; 4 Biostatistics Department, Boston University School of Public Health, Boston, MA 02118, USA; 5 Department of Clinical Immunology, Nova Southeastern University, 3200 South University Drive, Fort Lauderdale, FL 33328, USA; 6 Department of Cancer Biology and Pharmacology, University of Illinois College of Medicine Peoria, Peoria, IL 61605, USA; 7 Arnold School of Public Health, University of South Carolina, Columbia, SC 29208, USA

**Keywords:** Gulf War illness, microbiome, Gulf War, veterans, inflammation, cytokines, exposure

## Abstract

Gulf War Illness (GWI) is a chronic multi-symptom disorder affecting the central nervous system (CNS), immune and gastrointestinal (GI) systems of Gulf War veterans (GWV). We assessed the relationships between GWI, GI symptoms, gut microbiome and inflammatory markers in GWV from the Boston Gulf War Illness Consortium (GWIC). Three groups of GWIC veterans were recruited in this pilot study; GWV without GWI and no gastrointestinal symptoms (controls), GWV with GWI and no gastrointestinal symptoms (GWI-GI), GWV with GWI who reported gastrointestinal symptoms (GW+GI). Here we report on a subset of the first thirteen stool samples analyzed. Results showed significantly different gut microbiome patterns among the three groups and within the GWI +/−GI groups. Specifically, GW controls had a greater abundance of firmicutes and the GWI+GI group had a greater abundance of the phyla bacteroidetes, actinobacteria, euryarchaeota, and proteobacteria as well as higher abundances of the families Bacteroidaceae, Erysipelotrichaceae, and Bifidobacteriaceae. The GWI+GI group also showed greater plasma levels of the inflammatory cytokine TNF-RI and they endorsed significantly more chemical weapons exposure during the war and reported significantly greater chronic pain, fatigue and sleep difficulties than the other groups. Studies with larger samples sizes are needed to confirm these initial findings.

## 1. Introduction

Gulf War Illness (GWI), a debilitating multi-symptom illness, has affected and altered the quality of life for thousands of US Gulf War veterans (GWV). Approximately 700,000 troops from the US were deployed to the Persian Gulf and the estimates are that this illness afflicts a third of those who were deployed [1]. Shortly following the end of the Gulf War in 1991 veterans began reporting a constellation of health symptoms from multiple body systems [1,2]. Researchers began what has now become a multi-decade long quest to discover the causes of this illness. Over the years, substantial evidence has accumulated supporting a link between deployment to the Persian Gulf during Operation Desert Shield/Operation Desert Storm, environmental exposures from the war and the development of GWI-related symptoms [1,2,3,4,5]. More specifically, researchers have identified a link between specific toxicant exposures including pesticides, anti-nerve gas pills (pyridostigmine bromide, PB) and nerve agent chemical weapons (sarin/cyclosarin), during deployment and development of GWI-related health symptoms [1,2,3,4,5]. In addition, it has recently been shown that GW veterans have not gotten better over time and may be getting worse as well as developing more chronic health conditions at a younger age than the general population [6].

GWI is a chronic health disorder that involves multiple body systems and includes multiple health symptoms. The hallmark symptoms of GWI according to the widely used Kansas criteria are fatigue, musculoskeletal pain, cognitive difficulties, gastrointestinal issues (GI), respiratory problems and skin rash [2,7,8]. A growing body of evidence indicates that GWI is associated with diverse central nervous system (CNS) and immune alterations suggesting a neuroinflammatory component to the disorder, but the specific pathobiological processes driving the diverse GWI symptoms have not been clearly elucidated [1,9,10,11]. Animal studies indicate that a chronic CNS inflammatory state can develop in response to an insult—chemical injury, infection or physical trauma—that mobilizes the CNS defense systems via activation of glia, the brain’s primary immune response cells, and release of chemical messengers (i.e., cytokines) that precipitate a complex set of symptoms. These symptoms have been identified as impaired memory and learning, increased pain sensitivity and persistent fatigue, a symptom complex similar to that of GWI [10,11,12,13,14,15]. Recent studies have also demonstrated CNS inflammatory effects of GW-related exposures (PB, pesticides, depleted uranium, nerve agents) and additional innate immune processes (oxidative stress markers) that potentially explain the mechanism contributing to the full spectrum of GWI symptoms including those of the gastrointestinal (GI) system. Specifically, it is postulated that GWI can occur through activation of the innate immune system through toll like receptors (TLR4) present in the CNS and the GI tracts [10,11,12,16,17,18]. Another not mutually exclusive hypothesis for GWI centers on mitochondrial impairment as the impetus for the disorder [19,20,21].

Much of our research done to date on specific symptomology of GWI has focused on the CNS effects including cognitive and mood difficulties experienced by GW veterans. Only more recently has attention started to turn toward understanding the relationship between CNS effects and other debilitating symptoms including GI disturbances and chronic pain experienced by GW veterans. Veterans with GWI report experiencing numerous chronic GI symptoms including; abdominal pain/discomfort, bloating, nausea, vomiting, diarrhea as well as a clinical diagnosis of Irritable Bowel Syndrome (IBS) in many cases [22,23,24]. Our group has recently demonstrated the role of the gut microbiome in a mouse model of GWI on the gut microbiome-brain axis and the enteric nervous system [16,25,26]. This work revealed a potential link between the altered gut microbiota to gut leaching, GI inflammation, systemic endotoxemia, and neuroinflammation through activation of toll-like receptor 4 (TLR4) located on glial cells in the brain and the GI system [16]. TLR4 activation leads to production and signaling of proinflammatory cytokines. To date, the gut microbiome and its relationship with innate immune system activation and neuroinflammatory cytokine blood markers has not been examined in GW veterans.

In order to more fully assess the relationships between GWI, GI symptoms and potential innate immune activation and neuroinflammation in GW veterans, we translated our prior GW-relevant animal studies of microbiome and proinflammatory cytokines (i.e., gut microbiome-brain axis) into a pilot study with GW veteran participants from the Boston Gulf War Illness Consortium (GWIC) study. The Boston GWIC was designed to have both preclinical and clinical epidemiologic studies working in tandem to translate results as expediently as possible to the clinic. The goal of this GWIC funded microbiome call-back pilot study was to determine (1) whether the gut microbiome diversity of GW veterans with GWI and GI symptoms (GWI+GI) is different than the gut microbiome of GW veterans with GWI and no GI symptoms (GWI-GI) and/or different than GW veterans without GWI or GI symptoms (controls) and (2) whether these three groups differ with respect to innate immune system activation as shown by proinflammatory cytokine levels in circulating plasma and (3) whether these markers correlate with self-reported toxicant exposures from the war.

## 2. Materials and Methods

### 2.1. Human Subjects

The GWIC includes a series of clinical studies with the primary objective of providing a cohesive understanding of the pathobiological mechanisms responsible for the varied symptoms of GWI in order to provide a rational and efficient basis for identifying beneficial treatments and diagnostic markers. Inclusion criteria for GWIC required deployment to the Gulf War between August 1990 and July 1991. Exclusion criteria included chronic illness diagnoses that could otherwise account for the symptoms endorsed by veterans. These included autoimmune, central nervous system, or major psychiatric disorders that could affect brain and immune functions (e.g., epilepsy, stroke, severe head injury, brain tumor, multiple sclerosis, Parkinson’s disease, Alzheimer’s disease, schizophrenia, bipolar disorder, and autoimmune disorders). The Kansas GWI case criteria required endorsement of at least 3 out of 6 symptom domains (fatigue, pain, neurological, skin, gastrointestinal, and respiratory) of either at least moderate severity or multiple mild symptoms within the domain [7]. GWIC participants not meeting Kansas GWI or exclusionary criteria were considered controls.

To date, the GWIC has recruited and examined over 230 GW veterans. Each of these veterans has undergone an extensive assessment, including numerous health-related surveys, a neuropsychological test battery, brain imaging and collection of blood and saliva samples [27]. Specifically related to the gastrointestinal tract, subjects were asked to report whether they experienced the following symptoms in the past 6 months: abdominal pain or cramping, diarrhea, nausea or upset stomach, other non-specified GI disorders. In addition, they were asked to report whether they had received a diagnosis of IBS from a doctor as part of the medical conditions questionnaire. The GWIC study was awarded exploratory funds to perform promising pilot studies to be used as preliminary data for future larger studies. One of the approved pilot studies was the current GWIC call back study of microbiome patterns in GW veterans. Thirty GW veterans who had participated in the GWIC study were targeted for further participation in this microbiome pilot study.

For this pilot study three groups of veterans from the GWIC cohort were recruited including: 1. GW veterans without GWI who reported no gastrointestinal symptoms/disorder (GW controls), 2. Gulf War veterans with GWI who reported no gastrointestinal symptoms/disorders (GWI-GI) and 3. Gulf War veterans with GWI who reported gastrointestinal symptoms/disorders (GW+GI).

Participants for this pilot call back study were recruited by telephone after they completed the GWIC study protocol and asked to fill out a brief subject screening questionnaire and provide a stool sample by mail to the study investigators. Once the subjects received the stool collection kit they were asked to collect the sample at their homes at their own convenience and mail back to Boston University GWIC investigators.

### 2.2. Demographics, Deployment Exposures and Health Symptom Surveys

GWIC subjects were administered a general demographic information and medical conditions questionnaire, the Kansas Gulf War and Health Questionnaire and Kansas Gulf War Experiences and Exposure Questionnaire, and the Structured Neurotoxicant Assessment Checklist (SNAC) [7,28,29]. Self-reported exposures were obtained from the Kansas GW Experiences and Exposure Questionnaire and the SNAC [7,28]. Health outcomes were measured by the medical conditions questionnaire in which the participant endorsed whether or not they had a confirmed diagnosis of queried health outcomes including memory loss, chronic fatigue syndrome, fibromyalgia and IBS [28]. Additional validated health symptom surveys were completed by study participants including the Multi-dimensional Fatigue Inventory (MFI-20), McGill Pain Inventory and the Pittsburgh Sleep Quality Index where higher scores indicated more symptoms [30,31,32].

### 2.3. Plasma Cytokine Analyses

Plasma was separated and stored at −80 °C until assayed. An 18 cytokine Quansys multiplex panel was used to measure cytokines in plasma samples in the GWIC study. The proinflammatory cytokines including IL1α and IL1β, and soluble receptors for TNF Receptor I (TNF-RI), TNF Receptor II (TNF-RII) as well as Th1, Th2, Th17 and anti-inflammatory cytokines were measured with a multiplex chemiluminescent assay using Quansys instrument and reagents in methods previously reported [33]. To determine if circulating proinflammatory cytokines levels were different across the three groups in this pilot study, plasma samples were examined by symptom group. In this study, chemiluminscent imaging concentrations of IL-1a, 1b, 2, 4, 5, 6, 8, 10, 12 (p70), 13, 15, 17 and 23, IFNγ, TNFα, TNFβ, TNF-RI and TNF-RII in plasma samples were examined.

### 2.4. Stool Sample Collection

Subjects were mailed a stool collection kit, a Second Genome (Second Genome, San Fransisco, CA, USA) stool collection vial, a pre-paid envelope to return the collection vial and a questionnaire pertaining to antibiotic use over the past 6 months. The Second Genome’s stool collection vial is a self-collect, high-quality stool sample at home kit. Each collection vial was barcoded and added with nucleic acid stabilizing solution. This aids in the rapid homogenization and stabilization of DNA/RNA at the time of collection and protects high-quality samples during transport and long-term storage. Veterans were asked to collect their stool and then transfer a small amount of stool into the vial. This vial was then placed in a zipper pouch with absorbent material in case of spills. The zipper pouch was then placed into a plastic bubble wrap shipping envelope and mailed to investigators at Boston University School of Public Health. Once received the stool sample was stored at −20 °C and then shipped to collaborators at the University of South Carolina (USC) for analysis. Samples were stored at −20 °C at the USC facility until a significant number of samples were received from each of three study groups. Further, samples were shipped to Second Genome facility (Second Genome, South San Francisco, CA, USA) for further processing and analysis of gut microbiome.

### 2.5. Gut Microbiome Analysis

#### 2.5.1. Sample Isolation

Second Genome performed nucleic acid isolation with the Qiagen MagAttract PowerMicrobiome(Qiagen, Germantown, MD, USA) DNA/RNA Kit according to the manufacturer’s guidelines and optimized for high-throughput processing. All samples were quantified via the Qubit^®^ Quant-iT dsDNA High Sensitivity Kit (Invitrogen, Life Technologies, Grand Island, NY, USA) to ensure that they met minimum concentration and mass of DNA.

#### 2.5.2. Library Preparation

To enrich the sample for bacterial 16S V4 rDNA region, DNA was amplified utilizing fusion primers designed against the surrounding conserved regions which are tailed with sequences to incorporate Illumina (Illumina, Inc, San Diego, CA, USA) adapters and indexing barcodes. Each sample was PCR amplified with two differently barcoded V4 fusion primers and PCR products were quantified by fluorometric method (Qubit or PicoGreen from Invitrogen, Life Technologies, Grand Island, NY, USA). Samples that met the post-PCR quantification minimum were pooled equimolar and advanced for sequencing.

#### 2.5.3. Profiling Method

A pool containing 16S V4 enriched, amplified, barcoded samples were loaded into a MiSeq^®^ (Illumina, Inc, San Diego, CA, USA) reagent cartridge, and then onto the instrument along with the flow cell. After cluster formation on the MiSeq instrument, the amplicons were sequenced for 250 cycles with custom primers designed for paired-end sequencing. QC and QA metrics are maintained for all sample handling, processing, and storage procedures.

#### 2.5.4. Data Analysis

The microbiome sequence and data analysis were carried out at several separate stages: pre-processing, summarization, normalization, alpha diversity metrics (within-sample diversity), beta diversity metrics (sample-to-sample similarity), ordination/clustering, sample classification, and significance testing. For all analyses, *p* < 0.05 was considered statistically significant. Second Genome’s analysis software package was used for statistical analysis of microbiome diversity. For descriptive purposes, one-way ANOVA tests and Chi-square were used as appropriate, to compare symptom groups. For these analyses, SAS 9.4 (Statistical Analysis Systems, SAS Institute, Cary, NC, USA) was used.

This study was approved by the Boston University Medical Campus Institutional Review Board and well as the USAMRDC: Human Research Protection Office (HRPO).

## 3. Results

### 3.1. Human Subjects

#### 3.1.1. GWIC Full Study Cohort

Of the 230 GW veterans recruited into GWIC, 196 met Kansas criteria for GWI and 34 did not meet Kansas GWI criteria or Kansas exclusionary criteria and were considered healthy controls. Gastrointestinal symptoms are a prominently reported problem in veterans with GWI, however, they are not seen in all cases. Sixty-two percent of veterans with GWI reported abdominal pain or cramping, sixty-six percent reported diarrhea, sixty-two percent reported nausea or upset stomach and forty-eight percent reported being diagnosed with IBS. In comparison, in our control group 9% percent of veterans reported abdominal pain or cramping, 12% percent reported diarrhea, 18% reported nausea or upset stomach and 12% reported being diagnosed with IBS.

#### 3.1.2. GWIC Microbiome Pilot Call Back Study Cohort

Of the 30 participants targeted, a total of 27 GWV agreed to participate to date in the GWIC pilot call back study and provided a stool sample for analysis. In Group 1 (GW controls) there were 7 subjects, in Group 2 (GWV with GWI and no GI symptoms; GWI-GI) there were a total of 5 subjects, Group 3 (GWV with GWI and GI symptoms; GWI+GI) there were 14 subjects. Of those, only a subset has had their stool samples analyzed to determine their gut microbiota. In Group 1, there were 5 subjects, Group 2 included 3 subjects and Group 3 included 5 subjects. There were no statistically significant differences seen between the groups on demographic characteristics, p-values ranged from 0.23–0.95 (Table 1). There were also no statistically significant differences seen between the groups on Body Mass Index (*p* = 0.664) or self-reported high sugar or diabetes (*p* = 0.420) (Table 1). We examined self-reported exposures in theater and found a statistically significant difference in the groups for self-reported exposure to chemical/biological weapons (Table 1). The majority (80%) of cases with GWI+ GI experienced chemical weapons exposure, 66.7% of those with GWI–GI and none of the controls experienced chemical weapons exposure. There were no statistically significant differences among the groups with respect to exposure to PB anti-nerve gas pills, pesticide treated uniforms or being present in an area that had been fogged or sprayed with pesticides (Table 1). There were significant differences among the groups with regard to pain, fatigue and sleep problems with the GWI+GI group reporting far more symptoms on the McGill Pain score, Multi-dimensional Fatigue Inventory (MFI-20) and the Pittsburgh Sleep Quality Index (PSQI) than the GWI-GI or the control group (Table 1).

### 3.2. Cytokine Results

To determine if circulating proinflammatory cytokines levels were different across the three pilot study groups, plasma samples were collected and examined. Using chemiluminscent imaging concentrations of IL-1a, 1b, 2, 4, 5, 6, 8, 10, 12 (p 70), 13, 15, 17 and 23, IFNγ, TNFα, TNFβ, TNF-RI and TNF-RII in plasma samples were examined. No statistically significant differences were found among the groups for the following plasma cytokine concentrations: IL-1a, 1b, 2, 4, 5, 6, 8, 10, 12 (p 70), 13, 15, 17 and 23, IFNγ, TNFα, TNFβ, and TNF-RII (*p*-values ranging from 0.06–0.69). However, we found a statistically significant difference among the groups in circulating levels of TNF RI which plays a role in regulating inflammation (*p* = 0.04), such that GW controls had lower levels (452.6 ± 183.1) than GWI-GI (819.2 ± 250.3) and GWI+GI groups (852.7 ± 244.0) (Table 2).

### 3.3. Gut Microbiome Results

#### 3.3.1. Sample Diversity

To assess differences in the number of different species across the three groups, species richness was calculated using OTU richness (number of operational taxonomic units). Species richness was significantly lower in the GW controls (Mean = 415 ± 83.1) than in the GWI-GI (Mean = 576 ± 12.9) (*p* = 0.03), and GWI+GI (Mean = 501 ± 56.4). Shannon index was used to determine the evenness or balance of distribution of microbiome species across groups. GW controls had lower Shannon diversity scores (3.79 ± 0.226) than GWI-GI (4.03 ± 0.147) and GWI+GI groups (3.94 ± 0.163) (Table 3).

#### 3.3.2. Phyla Distribution

**Figure 1:**
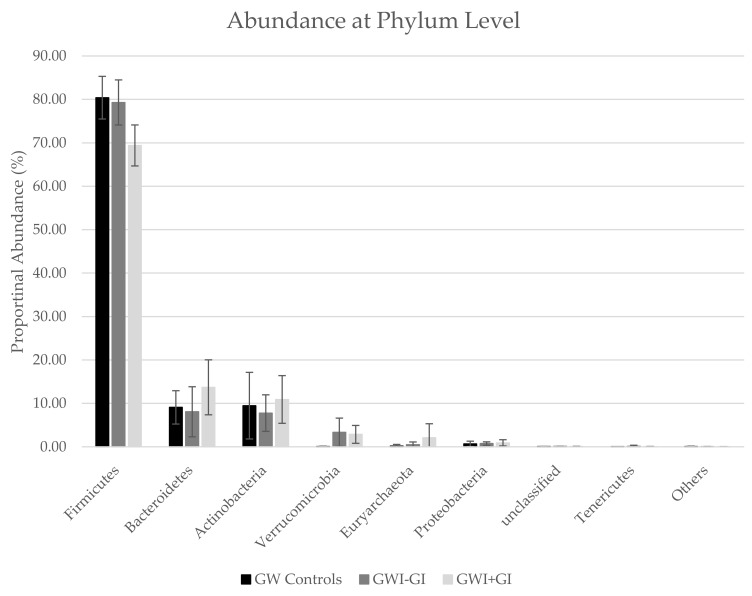
Proportional Abundance at the Phylum and Family Level by Group.

Firmicutes was the most abundant phylum across all groups (Table 4, Figure 1). A significant increase was observed in the relative abundance of Firmicutes in the GW controls (80.4 ± 4.91) and GWV with GWI-GI symptoms (79.3 ± 5.19; *p* = 0.03) compared to GWV with GWI+GI symptoms (69.4 ± 4.72; *p* = 0.01). We also observed higher abundance of Bacteroidetes, Actinobacteria, and Euryarchaeota in GWI+GI (13.7 ± 6.33; 10.9 ± 5.49; and 2.09 ± 3.22) than in GW controls (9.08 ± 3.84; 9.47 ± 7.67; and 0.17 ± 0.38) or GWI- GI (8.06 ± 5.77; 7.76 ±4.21; and 0.748 ± 0.391).

#### 3.3.3. Family Distribution

**Figure 2:**
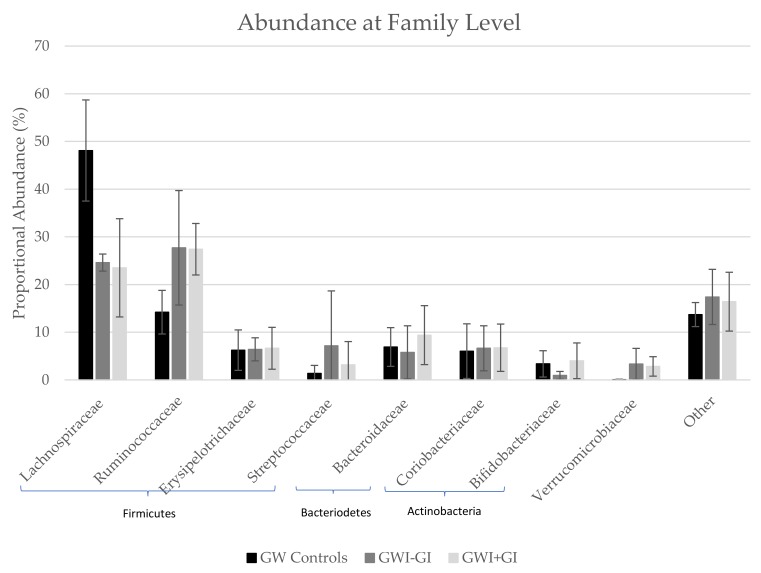
Proportional Abundance at the Phylum and Family Level by Group.

At the family level, Lachnispirache was the most abundant family in the GW controls but only the second most abundant in GWI-GI and GWI+GI, where Ruminococcaceae was the most abundant (Table 5, Figure 2). A significant increase was observed in the relative abundance of Lachnispiracae in GW controls (48.1 ± 10.6) compared to GWI-GI (24.6 ± 1.79; *p* = 0.03) and GWI+GI (23.5 ± 10.3; *p* = 0.03). GWI+GI had statistically significantly higher abundance of Ruminococcaceae (27.4 ± 5.4) than GW controls (14.2 ± 4.57) and approximately the same abundance as GWI-GI (27.7 ± 12). Bacteriodaceae and Bifidobacteriaceae were also more abundant in GWI+GI (9.39 ± 6.18 and 4.01 ± 2.04) than GW controls (6.9 ± 4.06 and 3.36 ± 2.76).

#### 3.3.4. Genus Level

A number of genus were more abundant in GW controls than GWI+GI and GWI-GI. Dialister was statistically significantly enriched in GW controls compared to GWI-GI (*p* = 0.008). Roseburia was statistically significantly enriched in GW controls compared to GWI+GI (*p* = 0.001). Ruminococcus was statistically significantly more abundant in GWI-GI and GWI+GI compared to GW controls (*p* = 0.038 and *p* = 0.007). Dialister was statistically significantly less abundant in GWI-GI compared to GW controls and GWI+GI (*p* = 0.008 and *p* = 0.003).

## 4. Discussion

The findings from this small pilot study suggest that the gut microbiome is significantly different in veterans with GWI+/-GI and GW veteran controls. There also appears to be a significant difference between the GWI+GI group and GWI-GI group with regard to microbiome diversity. Specifically, we found that GW controls had a greater abundance of firmicutes, including the family lachnospiraceae and the genus Dialister and Roseburia compared to GWI+GI and GWI-GI. In addition, compared to GW controls and veterans with GWI+GI, veterans with GWI-GI had a higher abundance of verrucomicrobia, and verrucomicrobiaceae as well as higher abundances of the families ruminococcaceae and streptococcaceae. The GWI+GI group had a greater abundance of the phyla bacteroidetes, actinobacteria, euryarchaeota, and proteobacteria as well as higher abundances of the families Bacteroidaceae, Erysipelotrichaceae, and Bifidobacteriaceae when compared to the groups of GW control veterans and GWI-GI veterans.

A study by Rizzatti et al. [34] found an association between proteobacteria and gut inflammation and Inflammatory Bowel Disease (IBD) such as Crohn’s disease and ulcerative colitis. In our study, we found a higher abundance of proteobacteria in our GWI+GI and GWI-GI compared to our GW controls. In this pilot study, we found a higher abundance of genus Roseburia in the GW controls compared to those with GWI, both with and without GI symptoms. Researchers have found that individuals with IBS have less abundance of Roseburia when compared to controls [35]. Roseburia has been shown to help digest complex carbohydrates [36] and also aid in the production of butyrate ([37]. Butyrate (butyric acid) is a short-chain fatty acid and its beneficial effect has been recently discussed in a preclinical study of colitis and IBD [38]. In our recent investigation, sodium butyrate priming improved gut microbial dysbiosis, intestinal epithelial membrane integrity, and intestinal inflammation in a PB and permethrin exposed mouse model of GWI [26]. A separate PB-exposed animal model of GWI by another research group also recently reported intestinal glial inflammation and chronic brain glial activation [17]. Although PB pill usage and permethrin treated uniforms were reported more commonly in GWI+GI group, only self-reported exposure to chemical warfare agents (sarin) was significantly different among the groups with over three-quarters of the GWI+GI group reporting this exposure during deployment and none of the GWI-GI or controls. This suggests an additional exposure target for preclinical etiological and treatment studies of GWI+GI symptoms.

In this pilot study, a decrease in the abundance of Roseburia in GWI+GI and GWI-GI, might be a novel cause of gastrointestinal inflammation in GWI. Correspondingly, our study also found significantly increased circulating levels of the proinflammatory cytokine TNF-RI in plasma from our GWI+GI group. The levels of TNF-RI were nearly double that of controls in the GWI+GI and GWI-GI groups suggesting significantly more inflammatory signaling in the GWI groups. These results correspond with recent therapeutic targets for IBD which focus on anti-TNF therapies to reduce gut inflammation associated with the disorder including those targeted on TNF-RI specifically [39].

Additional important correlations with findings in the literature and our findings include a less abundance of Dialister and the association with depression [40] as well as arthritis [41] and the positive association between Ruminococcus and IBS [42] and lupus [43]. In our pilot study, we found that the GWI+GI group reported much higher symptom burden for pain (McGill Pain Scale), fatigue (MFI-20) and sleep (PSQI) on validated measures compared with both the GWI-GI and control groups. These finding also correspond with prior studies reporting lower pain thresholds in GW veterans with GI in other study cohorts suggesting that a targeted approach of treating inflammatory mediators in GWI+GI could potentially reduce other chronic health symptoms [22,23,24].

As with all studies, this study had both strengths and weaknesses. To our knowledge, this is the first study conducted in a population of Gulf War veterans aimed at examining their gut microbiome and innate immune markers. As a follow-on study to the larger Gulf War Illness Consortium, we were able to recruit a number of veterans in a short amount of time who agreed to provide us with stool samples. In addition, we were able to separate our veterans into three clearly distinct groups based on GWI and GI symptoms. A major limitation of this study is the small sample size of a pilot study resulting in low statistical power to detect differences among the groups. Additionally, we were limited in how far down the taxonomic level we were able to analyze using the current methods employed. We also may not have had the power in this small sample to detect smaller cytokine effects among the groups (IL1, TNF-alpha, IL8) that have also been previously associated with IBD. Correspondingly, due to small sample size it was not possible to assess the impact of multiple exposures (i.e., PB and chemical weapons or depleted uranium) on gut microbiome and inflammatory markers. In addition, statistical analysis was limited in this pilot study with multiple testing and no post-hoc correction which could result in Type I errors. Some veterans with GWI have been reported to experience increased infections and could have increased exposure to antibiotics which could alter their gut microbiome. Participants in our study did not use antibiotics during the two weeks prior to stool collection but future studies should get a detailed history of antibiotic use in the veterans for a longer period of time.

Recent studies on human gut microbiome emphasize its association with a variety of diseases including obesity, diabetes type 2 (metabolic disorder), inflammatory bowel disease, liver disease, cancer, and neurological disorders [44,45,46,47]. The findings from this pilot study and our recent mouse model of GWI showed both similarities and discrepancy in the microbiome composition [16]. Interestingly, in both studies’ Firmicutes and Bacteroidetes remain two major phyla of the gut microbiota. Firmicutes was decreased and Bacteroidetes was increased in human microbiota of the GWI+GI group, however, Firmicutes was increased and Bacteroidetes was decreased in mouse microbiota of GWI. A closer look at both microbiome compositions suggest that the family Ruminococcaceae has been significantly increased in both the human and mouse microbiome. Other research by Ley et al. [48] suggest that though the two dominating phyla are Firmicutes and Bacteroidetes, at lower taxonomic classification the microbiome composition differ by 85% in healthy human and mouse [49]. The variation between human and mouse microbial composition may be due to the limitations in 1) diet control (mouse model has a similar diet composition; however, human diet composition varies), sample collection (in the mouse model mainly luminol or fecal content are collected, however, in the human fecal matter are collected) and analysis methods (mouse microbiome are limited to 16s rDNA sequencing, however, human microbiome analyzed by 16s rDNA or metagenomics). As designed, our GWIC study infrastructure provided the opportunity for the initial translation of our GWI animal microbiome study results to clinical relevance in this pilot study that will lead the way for further larger, more definitive studies.

Future directions of this work should include studies that build upon these initial provocative findings and increase the sample size to further validate these initial findings. In addition to increasing the overall sample size, it would be informative to add an additional IBS or IBD non-GWV control group and additional mixed exposure GWI groups. Additional work should be done to examine the peripheral markers of inflammation to examine the link between the altered gut microbiome and blood markers of inflammation, including additional TLR4 circulating markers such as HMGB1 recently found to be associated with animal models of GWI [12]. The gut-microbiome-brain axis could also be examined further by studying the association between gut microbiome changes, neuroinflammatory signaling and cognitive and neuroimaging findings in GW veterans. With larger sample sizes, it will also be possible to assess the contribution of combination exposures (PB, pesticides, chemical weapons and depleted uranium). Finally, it is possible to alter the gut microbiome through treatments, both prescription and over-the-counter, and the next direction for treatment possibilities for ailing GW veterans could be to examine the efficacy and safety of treatment approaches that target the gut microbiome as a novel treatment approach for the varied symptoms of GWI.

## 5. Conclusions

The findings from this small pilot study suggest that the gut microbiome is significantly different in veterans who have GWI and those who do not have GWI. There also appears to be a significant difference between the GW veterans with GWI and GI disturbance and the GW veterans with GWI but no GI disturbance with regard to microbiome diversity. The GWI+GI group also showed greater plasma levels of the inflammatory cytokine TNF-RI and they endorsed significantly more chemical weapons exposure during the war and reported significantly greater chronic pain, fatigue and sleep difficulties than the other groups. Studies with larger samples sizes are needed to confirm these initial findings.

## Figures and Tables

**Table 1:** Demographic and Exposure Characteristics of Participants.

Characteristic	GW Control *n* = 5	GWI-GI *n* = 3	GWI+GI *n* = 5	*p*-Value
Age, years [Mean (SD ^1^)]	52.8 (6.7)	63.2 (15.5)	53.3 (7.2)	0.296
Height, in [Mean (SD ^1^)]	69.8 (1.0)	67.5 (4.4)	66.8 (2.7)	0.238
Weight, lbs [Mean (SD ^1^)]	198.5 (18.3)	207.0 (24.3)	217.0 (99.7)	0.904
Gender [N (%)]				0.420
Male	5 (100)	2 (66.7)	4 (80)	
Female	0 (0)	1 (33.3)	1 (20)	
Race/Ethnicity [N (%)]				0.420
Black	0 (0)	0 (0)	1 (20)	
White	5 (100)	3 (100)	4 (80)	
Education [N (%)]				0.482
High School plus other training (technical/trade)	1 (20)	0 (0)	0 (0)	
Associates Degree or 2 years of College	0 (0)	0 (0)	2 (40)	
Some College	0 (0)	0 (0)	1 (20)	
Bachelor’s Degree	2 (40)	1 (33.3)	0 (0)	
Advanced or Professional Degree	1 (20)	2 (66.7)	2 (40)	
Highest Grade [Mean (SD ^1^)]	15 (2)	15 (1)	16 (3)	0.957
				
McGill Pain Score [Mean (SD)]	12 (8)	32 (7)	39 (16)	0.021
				
Multi-dimensional Fatigue Inventory (MFI-20) [Mean (SD)]	40 (8)	53 (7)	72 (10)	0.0005
				
Pittsburgh Sleep Quality Index (PSQI) [Mean (SD)]	8 (3)	7 (2)	14 (2)	0.003
Body Mass Index (BMI) [Mean (SD)]	28.6 (2.5)	31.9 (0.7)	34.0 (14.2)	0.664
Self-reported high sugar or diabetes [N (%)] No Yes	5 (100.00)0 (0.0)	2 (66.7)1 (33.3)	4 (80.0)1 (20.0)	0.420
Exposed to chemical or biological warfare agents during military service [N (%)]				0.049
No	2 (40.0)	1 (33.3)	0 (0.0)	
Yes	0 (0.0)	0 (0.0)	4 (80.0)	
Not Sure	3 (60.0)	2 (66.7)	1 (20.0)	
Taken pyridostigmine bromide (anti-nerve agent) pills [N (%)]				0.420
No	1 (20.0)	1 (33.3)	0 (0.0)	
Yes	4 (80.0)	2 (66.7)	5 (100.0)	
Wore a uniform treated with pesticides [N (%)]				0.120
No	5 (100.0)	2 (66.7)	2 (40.0)	
Yes	0 (0.0)	1 (33.3)	3 (60.0)	
Saw the area in which you lived fogged or sprayed with pesticides [N (%)]				0.228
No	3 (60.0)	3 (100.0)	2 (40.0)	
Yes	2 (40.0)	0 (0.0)	1 (20.0)	
Not Sure	0 (0.0)	0 (0.0)	2 (40.0)	

^1^ SD standard deviation.

**Table 2:** TNF-RI and RII cytokine comparisons by GI symptom groups.

	GW Controls		GWI–GI		GWI+GI	
Cytokine	Mean	SD	Mean	SD	Mean	SD
TNF-RI *	452.6	183.1	819.2	250.3	852.7	244.0
TNF-RII	721.2	79.6	876.3	62.7	541.6	122.3

* groups significantly different at *p* < 0.05.

**Table 3:** Sample Diversity Results by Groups.

	GW Controls		GWI–GI		GWI+GI	
Family	Mean	SD	Mean	SD	Mean	SD
OTU Richness	415	83.1	576	12.9	501	56.4
Shannon Index	3.79	0.23	4.03	0.15	3.94	0.16

**Table 4:** Percent relative abundance at the Phyla Level by Groups.

	GW Controls		GWI–GI		GWI+GI	
Phylum	Mean	SD	Mean	SD	Mean	SD
Firmicutes	80.40	4.91	79.30	5.19	69.40	4.72
Bacteroidetes	9.08	3.84	8.06	5.77	13.70	6.33
Actinobacteria	9.47	7.67	7.76	4.21	10.90	5.49
Verrucomicrobia	0.05	0.09	3.35	3.26	2.86	2.06
Euryarchaeota	0.17	0.38	0.49	0.63	2.09	3.22
Proteobacteria	0.68	0.61	0.75	0.39	0.91	0.72
unclassified	0.08	0.02	0.10	0.02	0.09	0.02
Tenericutes	0.00	0.00	0.13	0.22	0.02	0.06
Others	0.06	0.10	0.04	0.03	0.02	0.02

**Table 5:** Percent relative abundance at the Family Level by Groups.

	GW Controls		GWI–GI		GWI+GI	
Family	Mean	SD	Mean	SD	Mean	SD
Lachnospiraceae	48.10	10.60	24.60	1.79	23.50	10.30
Ruminococcaceae	14.20	4.57	27.70	12.00	27.40	5.40
Erysipelotrichaceae	6.24	4.24	6.41	2.42	6.63	4.39
Streptococcaceae	1.37	1.67	7.16	11.50	3.17	4.87
Bacteroidaceae	6.90	4.06	5.78	5.57	9.39	6.18
Coriobacteriaceae	6.03	5.73	6.63	4.72	6.75	4.96
Bifidobacteriaceae	3.36	2.76	0.95	0.83	4.01	3.74
Verrucomicrobiaceae	0.05	0.09	3.35	3.26	2.83	2.04
Other	13.70	2.52	17.40	5.78	16.40	6.17
